# Association of Sociodemographic Characteristics with the Time Spent in Occupational Activities of Older Persons in Malaysia

**DOI:** 10.21315/mjms-07-2025-563

**Published:** 2025-12-31

**Authors:** Ker Ke Jun, Dzalani Harun, Siti Fairuz Ismail, Nor Diana Abdul Rahim, Hanif Farhan Mohd Rasdi, Ismarulyusda Ishak

**Affiliations:** 1 Centre for Rehabilitation and Special Needs Studies, Occupational Therapy Programme, Faculty of Health Sciences, Universiti Kebangsaan Malaysia, Kuala Lumpur, Malaysia; 2 Centre for Toxicology and Health Risk Research, Faculty of Health Sciences, Universiti Kebangsaan Malaysia, Kuala Lumpur, Malaysia

**Keywords:** older persons, occupational activities, occupational engagement, sociodemographic factors, activity centres for older persons

## Abstract

**Background:**

The rate of population ageing is increasing far more quickly than in the past, making active ageing a critical focus. Occupational activities are the daily tasks performed by individuals and are closely related to well-being. Research on sociodemographic factors associated with the time spent in occupational activities by older persons in Malaysia is limited. Understanding these patterns can help policymakers and healthcare providers tailor interventions to support different demographic groups among older persons. The occupational activities examined in this study were personal care, employment-related, education, domestic, childcare, purchasing, voluntary, social, and leisure activities. The objective of this study was to identify the differences in the time spent by older persons in occupational activities based on sociodemographic factors.

**Methods:**

A cross-sectional study involving 270 older persons from activity centres for older persons in Klang Valley was conducted using the Modified Occupational Questionnaire-Malay version and analysed using Mann-Whitney U test and Kruskal–Wallis test.

**Results:**

Findings show significant differences in time spent on domestic activities based on employment status (*P* = 0.046), employment-related activities based on type of housing and household size (*P* = 0.034; *P* = 0.047), and leisure activities based on household size (*P* = 0.006), while social activities differed based on gender (*P* = 0.038) were observed. Working older persons engaged more in domestic activities than non-working older persons; women engaged more frequently in social activities than men. A significant difference was also found in the time spent on employment-related activities between living in high-end and affordable housing.

**Conclusion:**

Tailoring interventions based on these factors is crucial for promoting active ageing and enhancing the well-being of older persons.

## Introduction

According to the Institute for Public Health (IPH), National Institutes of Health, Ministry of Health Malaysia (2018), pre-older persons refer to those aged 50 to 59 years, and older persons refer to those aged 60 years and above (1). In 2024, the population aged 60 years and above in Malaysia was approximately 3.9 million, accounting for 11.6% of the total population (2). The number and proportion of older persons in Malaysia are increasing (3). By 2030, Malaysia is forecasted to be an ageing nation, with an estimated increase in the proportion of older persons reaching 15.3%, approximately 5.8 million (3). In 2040, it is projected to be 17.3%, accounting for 6.4 million older persons (4).

Government organisations have created several plans and initiatives to enhance and support older persons’ well-being and healthy ageing (5). The United Nations (UN) declared the Decade of Healthy Ageing (2021–2030) in 2020, in line with the 2030 Agenda for Sustainable Development Goals (6). There are 17 SDGs being actively worked on in Malaysia, and the third goal focuses on good health and well-being (6).

The term “occupational activity” refers to meaningful and individualised daily tasks performed by individuals (7). According to the Australian Bureau of Statistics (8), occupational activities listed in the Time Use Survey include personal care, employment activity, education, domestic, childcare, purchasing, voluntary and care, social and community, and leisure. Occupational activity is closely associated with an individual’s health and perception of well-being (7, 9). This emphasises the value of older persons’ occupational engagement in fostering a meaningful life and increasing their well-being.

Active ageing is closely linked to the engagement of older persons in occupational activities (10), and various sociodemographic factors influence the time spent in these activities (11–15). As Malaysia prepares for a rapidly ageing society, gaps remain in understanding how sociodemographic factors influence the engagement of older persons in occupational activities. Thus, this study aims to identify the differences in the time spent by older persons in occupational activities based on sociodemographic factors.

## Methods

### Study Design, Study Population and Sampling Method

The study population consisted of older persons at the Activity Centre for the Older Persons (Pusat Aktiviti Warga Emas [PAWE]) in the Klang Valley, under the Department of Social Welfare (Jabatan Kebajikan Masyarakat [JKM]). A total of 275 participants were recruited through convenience sampling. Five participants were excluded from the analysis because of incomplete forms, resulting in a final sample of 270 participants. The sample size was calculated using the formula of Krejcie and Morgan (16), as the population size in the PAWE involved was known (16). The formula used is as follows:


$$n=\frac{x^{2}NP(1-P)}{A^{2}(N-1)+x^{2}P(1-P)}$$

where: *x*^2^ = 3.84, *P* = 0.5, *A* = 0.05, and *N* = 718


$$\begin{array}{c} n=\frac{3.84\;(718)\;(0.5)\;(1-0.5)}{0.05^{2}\;(718-1)+3.84\;(0.5)\;(1-0.5)}+10\%\;\text{dropout}\;\text{rate}\\ n=2.75 \end{array}$$

The inclusion criteria for participants were those aged 50 years and above in Klang Valley and who could understand, read, and communicate in Malay. The cutoff age of 50 years, representing the pre-older persons group, was chosen because individuals at this age are also at an increased risk of physical inactivity, which affects their engagement in occupational activities, as reported by the IPH, National Institutes of Health, Ministry of Health Malaysia (1). The exclusion criteria for the participants were those who had vision or hearing impairments that could not be corrected by an assistive device and who were not Malaysian citizens were excluded.

### Instrumentation

#### Sociodemographic Characteristics of the Study Participants

The demographic data form was distributed to all participants. The sociodemographic form included their contact information (name and phone number), age, gender, ethnicity, employment status, individual income, household size, type of house they lived in, and their health status (number of morbidities).

#### Modified Occupational Questionnaire-Malay Version

The Modified Occupational Questionnaire (MOQ) is a time use assessment designed to collect information about the meaningful use of time spent the previous day (17). Participants reported what they did on a typical day in half-hour blocks from 5 am to midnight (17). The Modified Occupational Questionnaire-Malay version (MOQ-M) demonstrated an excellent item-level content validity index (I-CVI), with scores ranging from 0.90 to 1.00 (18). The internal consistency of the MOQ-M ranged from 0.84 to 0.99 (18).

### Procedure

Ethical approval for this study was obtained from the University Ethics Committee, along with formal approval from the Department of Social Welfare (JKM) to conduct data collection at PAWE. Participants were recruited based on the inclusion and exclusion criteria. Data collection was conducted face-to-face with the participants at PAWE. Each participant was provided with an information sheet, informed consent form, demographic data form, and the Modified Occupational Questionnaire-Malay version (MOQ-M). The purpose of the study and the voluntary nature of participation were explained to the participants. Written informed consent was obtained from all participants before the study began. Clear instructions were provided to the participants to ensure proper understanding and completion of the MOQ-M.

Descriptive analysis was used to determine the sociodemographic of the older persons. The normality of the data was tested using the Kolmogorov-Smirnov test (*P* < 0.05), skewness, and kurtosis values. Skewness and kurtosis values falling outside the acceptable range of ± 2 further indicated that the data were not normally distributed. As a result, non-parametric tests were selected for analysis. Mann-Whitney U test was used to compare the time spent by older persons in occupational activities by age group, gender, and employment status. The Kruskal–Wallis test was used to compare the time spent by older persons in occupational activities according to ethnicity, household size, type of house, income, and health status. For variables with statistically significant Kruskal–Wallis test results (*P* < 0.05), Dunn’s post hoc pairwise comparisons with Bonferroni correction were conducted to identify specific group differences. All statistical analyses were performed using the IBM Statistical Package for the Social Sciences version 29.0.1.0.

## Results

### Demographic Characteristics

Table 1 presents the demographic data of the 270 participants. The descriptive statistics show that the sample consisted of more women (80.37%) than men (19.63%). Most participants (88.89%) were aged 60 years and above, and 95.9% were Malay. Most participants were not working (90.37%). The largest groups were those living in households with five or more persons (27%) and two-person households (22.2%). Regarding housing type, most of them resided in flats (39.6%) or village houses (26.3%). Monthly income levels varied across the sample, with more than half of the participants (54.4%) reporting a monthly income of RM 1,000 or below, followed by 21.5% earning between RM 1,001 and RM 2,000. Concerning the health status, a considerable proportion (36.7%) reported having two morbidities.

### Time Spent on Occupational Activities Based on Sociodemographic Factors

The time spent by older persons in various occupational activities, namely personal care, employment activity, education, domestic, voluntary and care, social and community, childcare, purchasing, and leisure, was analysed across several sociodemographic factors: age group, gender, ethnicity, employment status, household size, type of house, income, and health status.

**Figure 1:**
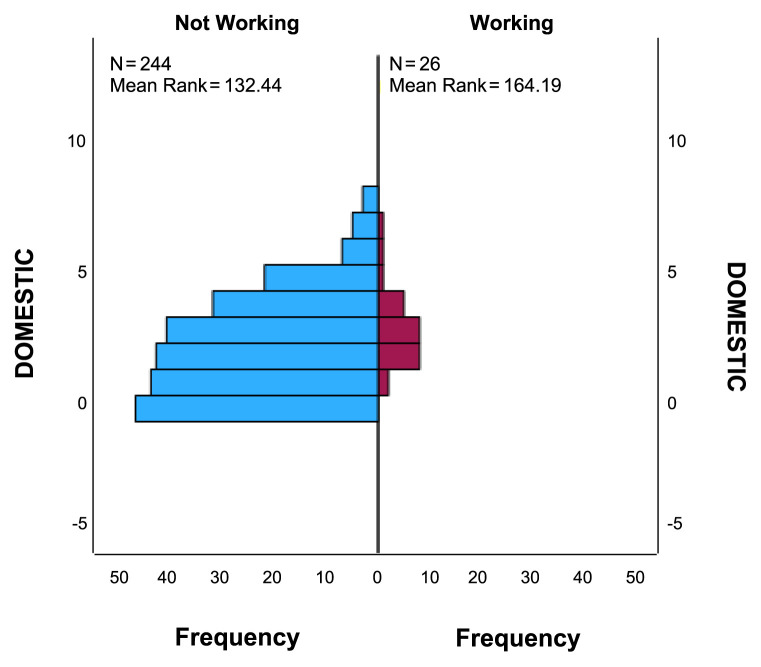
Time spent on domestic activities by older persons based on employment status

Several occupational activities showed significant differences based on sociodemographic factors. There was a significant difference in the time spent on domestic activities based on the employment status of older persons (*P* = 0.046). As illustrated in Figure 1, older persons who were employed (median = 3, IQR = 2) tended to spend more time on domestic activities compared with older non-working persons (median = 2, IQR = 3).

**Figure 2:**
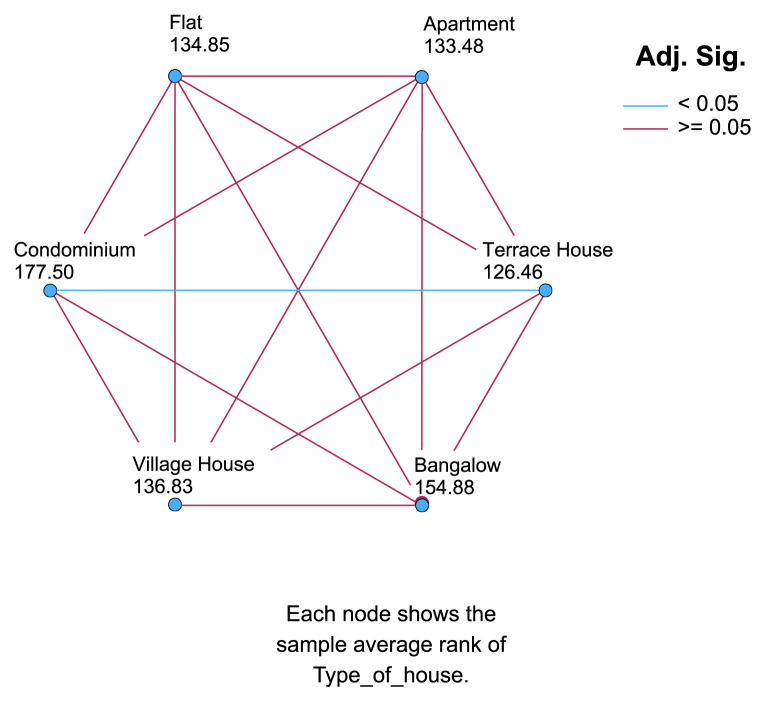
Time spent on employment-related activities by older persons based on the type of housing

Significant differences were also found in the time spent on employment-related activities based on housing type (*P* = 0.034) and household size (*P* = 0.047). Figure 2 presents the pairwise comparisons of the participants’ time spent in employment-related activities based on housing type. Pairwise comparisons revealed significant differences in the time spent on employment-related activities between those residing in condominiums (median = 0, IQR = 4) and those living in terraced houses (median = 0, IQR = 0), where condominium residents had a higher average rank (*r̄* = 177.50) than those in terraced houses (*r̄* = 126.46). This suggests that participants residing in condominiums spent significantly more time engaged in employment-related activities than those in terraced houses.

**Figure 3:**
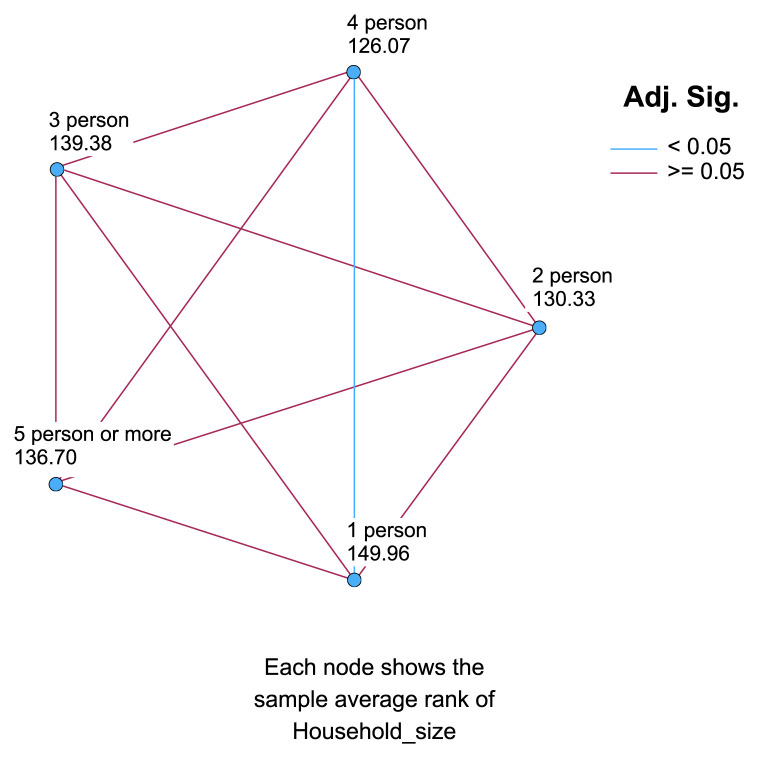
Time spent on employment-related activities by older persons based on household size

Figure 3 displays the pairwise comparisons of household size categories based on time spent in employment-related activities. A significant difference was observed between the one-person households (median = 0, IQR = 0) and four-person households (median = 0, IQR = 0) among all comparisons. The one-person household group exhibited the highest average rank (*r̄* = 149.96), suggesting greater engagement in employment-related activities, while the four-person group had the lowest average rank (*r̄* = 126.07).

**Figure 4:**
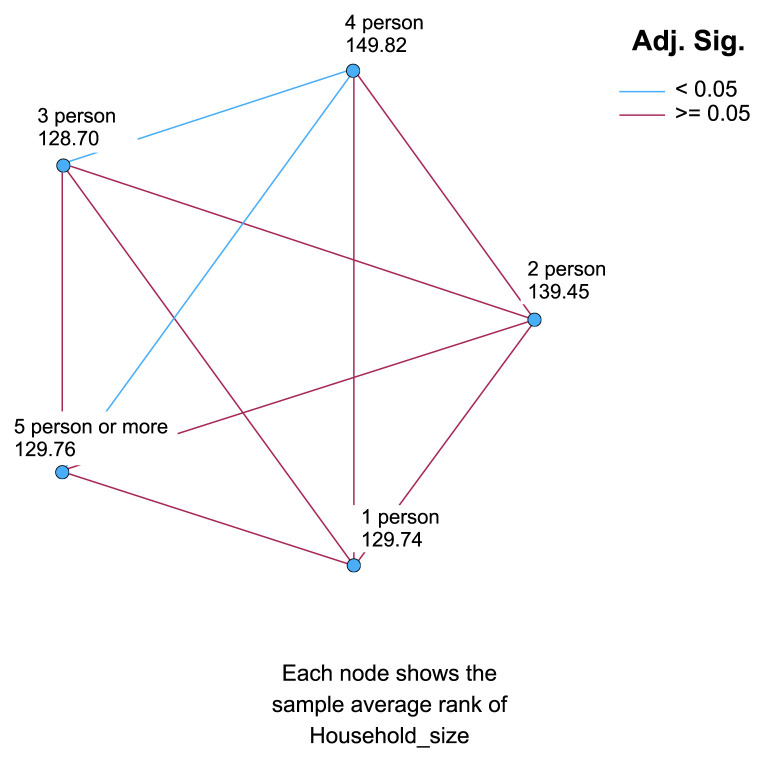
Time spent on leisure activities by older persons based on household size

Moreover, the time spent on leisure activities varied significantly across household sizes (*P* = 0.006). The pairwise comparisons shown in Figure 4 revealed that older persons living in four-person households (median = 0, IQR = 1) spent significantly more time engaged in leisure activities than those in three-person households (median = 0, IQR = 1) and those in households with five or more persons (median = 0, IQR = 2).

**Figure 5:**
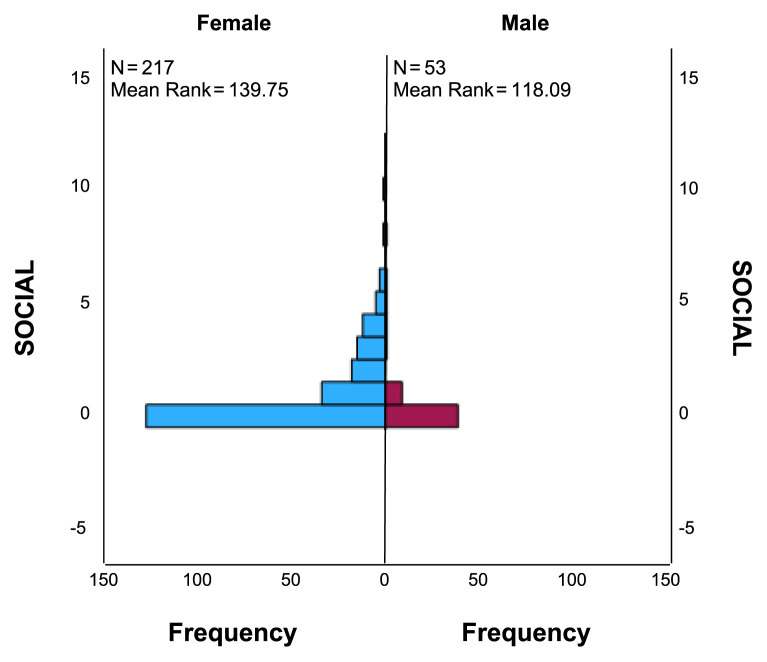
Time spent on social activities by older persons based on gender

Next, significant differences were also observed in social activity engagement based on gender (*P* = 0.038). As shown in Figure 5, females (median = 0, IQR = 2) spent more time in social activities than males (median = 0, IQR = 1). This indicates that older women engaged in significantly more social activities than older men.

## Discussion

This study highlights the time spent by older persons in occupational activities and how it differs according to sociodemographic factors. The findings of this study indicate that older persons who are employed tend to spend more time on domestic activities compared with those who are not. This finding contrasts with the research of Adjei and Brand (19), who observed that retired older persons allocate more time to domestic tasks than working older persons. However, it is also essential to consider that older persons, regardless of their employment status, tend to engage more frequently in passive and sedentary activities (20). These findings suggest that while employment status may influence the time spent by older persons on domestic activities, it does not necessarily change the overall trend toward sedentary lifestyles among older persons.

There was a significant difference in the time spent on employment-related activities between the residents of condominiums and those in terrace houses. Older persons living in condominiums appeared to dedicate more time to employment-related activities. Condominiums are generally considered a more luxurious form of housing in Malaysia (21), and owners usually must pay monthly maintenance fees. According to Rahayuwati et al. (22), older persons often continue to engage in paid work postretirement to address their financial needs. In contrast, terrace houses are the most common type of affordable housing, particularly among low-income groups such as the B40 category (23, 24). These factors may influence the allocation of time to employment-related activities in different housing settings.

Another notable difference in the time spent on employment-related activities was observed between households of various sizes, specifically between one-person and four-person households. Older persons who live alone need to keep themselves employed to meet their financial needs independently. Older persons who live with their family typically indicate that they have greater potential to receive financial support from their children (25) and reduce their time spent on employment-related activities. However, a study in Malaysia found that larger households may have weaker financial adequacy due to higher spending and lower savings (26). Therefore, the relationship between household size and time spent in employment-related activities among older persons is influenced by both support dynamics and overall household financial adequacy.

In addition, there was a significant difference in the time spent by older persons on leisure activities based on household size. Our result is consistent with the findings of Saidj et al. (27), who found that as household size increased, leisure time decreased. This may be attributed to increased caregiving responsibilities within larger households, especially in multigenerational living arrangements (28). Multigenerational living is defined as a household arrangement in which multiple generations of the same family, typically grandparents, parents, and children, stay together under one roof (28). Older persons in such households may prioritise family needs such as grandparenting, thereby reducing their leisure engagement (29).

Active engagement in social activities may reduce the risk of several age-related health conditions, improve health outcomes (30), and contribute to healthy ageing (31). The results of this study indicate that older women spend more time participating in social activities than older men. Thomas (32) indicated that older women who participate more in social activities have fewer physical and cognitive limitations later in life. Social participation is important for both physical and cognitive health. Furthermore, research has demonstrated that increased social participation reduces loneliness and improves the well-being of older persons (33). However, the social participation of older persons decreases with age for both genders (34). This has highlighted the need to encourage older persons to participate in social activities through community programmes or family support systems to improve their overall well-being.

Given Malaysia’s projected demographic shift toward an ageing population, the government must proactively address the needs of older persons. This need is further highlighted by the findings of Haidhir and Dahlan (29), who indicated that older persons in Malaysia have a stronger desire to engage in occupational activity than they are currently able to. Therefore, policymakers and healthcare providers can leverage these results to design targeted interventions or programmes that address the specific needs of diverse demographic groups among older persons (1), particularly within community-based settings such as the PAWE and other relevant organisations. Such initiatives can address the specific needs and challenges faced by older persons in Malaysia, ultimately enhancing their engagement in meaningful occupational activities and promoting holistic well-being.

### Limitation

This study has several limitations. First, the participants were older persons who lived in the community and did not include older persons who lived in institutions. Next, most of the older persons who participated in this study were Malay, which limits the representativeness of the findings for the broader Klang Valley population. To enhance the generalisability and provide a more comprehensive understanding of older persons in this region, future research should involve a more diverse sample of participants.

## Conclusion

Understanding the factors that influence older persons’ engagement in occupational activities has become increasingly important as Malaysia prepares for a rapidly ageing society. This study highlights that the time spent by older persons in occupational activities varies across several sociodemographic factors, including gender, employment status, household size, and type of housing. These findings underscore the importance of developing tailored interventions that consider these sociodemographic variables.

## Figures and Tables

**Table 1:** Sociodemographic characteristics of the participants

Sociodemographic characteristics	Frequency (*n* = 270)	Percentage (%)
Gender		
Male	53	19.6
Female	217	80.4

Age group		
50 to 59 years old	40	14.8
60 years old and above	230	85.2

Ethnicity		
Malay	259	95.9
Chinese	5	1.9
Indian	6	2.2

Employment status		
Working	26	9.6
Not working	244	90.4

Household size		
One person	35	13.0
Two persons	60	22.2
Three persons	51	18.9
Four persons	51	18.9
Five persons and above	73	27.0

Type of housing		
Flat	107	39.6
Apartment	26	9.6
Condominium	5	1.9
Village house	71	26.3
Terrace house	48	17.8
Bungalow	13	4.8

Income		
RM 1 to RM 1,000	147	54.4
RM 1,001 to RM 2,000	58	21.5
RM 2,001 to RM 3,000	29	10.7
RM 3,001 and above	36	13.3

Health status		
No morbidity	64	23.7
One morbidity	92	34.1
Two morbidities	99	36.7
More than three morbidities	15	5.6
